# Quantitative photoacoustic imaging using known chromophores as fluence marker

**DOI:** 10.1016/j.pacs.2024.100673

**Published:** 2024-12-19

**Authors:** Anjali Thomas, Max Rietberg, Mervenur Akkus, Gijs van Soest, Kalloor Joseph Francis

**Affiliations:** a Biomedical Photonic Imaging Group, Technical Medical Center, University of Twente, Enschede, 7522 NB, The Netherlands; b Erasmus MC, Cardiovascular Institute, Department of Cardiology, Biomedical Engineering, Rotterdam, The Netherlands; c Department of Precision and Microsystems Engineering, Delft University of Technology, Delft, The Netherlands; d Wellman Center for Photomedicine, Massachusetts General Hospital, Boston, USA

**Keywords:** Photoacoustic imaging, Tissue quantification, Optical inversion, Fluence correction, Fluence marker, Spectral imaging

## Abstract

Photoacoustic imaging offers optical contrast images of human tissue at acoustic resolution, making it valuable for diverse clinical applications. However, quantifying tissue composition via optical contrast remains challenging due to the unknown light fluence within the tissue. Here, we propose a method that leverages known chromophores (*e.g.*, arterial blood) to improve the accuracy of quantitative photoacoustic imaging. By using the optical properties of a known chromophore as a fluence marker and integrating it into the optical inversion process, we can estimate the unknown fluence within the tissue. Experimentally, we demonstrate that this approach successfully recovers both the spectral shape and magnitude of the optical absorption coefficient of an unknown chromophore. Additionally, we show that the fluence marker method enhances conventional optical inversion techniques, specifically (i) a straightforward iterative approach and (ii) a gradient-based method. Our results indicate an improvement in accuracy of up to 24.4% when comparing optical absorption recovery with and without the fluence marker. Finally, we present the method’s performance and illustrate its applications in carotid plaque quantification.

## 1 Introduction

Photoacoustic (PA) imaging emerges as a fast-growing technique in the biomedical imaging landscape, offering a unique blend of optical and ultrasonic modalities to visualize the light-absorbing structures and functional imaging of biological tissues [1]. PA imaging exploits the photoacoustic effect, where pulsed laser light induces thermoelastic expansion in tissues, generating ultrasound signals that are captured to construct high-resolution images [1]. The advancement of quantitative photoacoustic (QPA) imaging marks a significant leap forward, enabling not just visualization but also precise quantification of tissue chromophore and functions, such as hemoglobin concentration, oxygen saturation, and lipid imaging [2], [3], [4]. This quantitative aspect holds immense potential for clinical applications, ranging from oncology to cardiovascular and neurological imaging, where accurate, non-invasive insights into tissue physiology and pathophysiology can guide early diagnosis, treatment planning, and monitoring [5], [6]. Two key applications to list are plaque quantification in blood vessels [7] and oxygen saturation imaging [8], [9]. QPA algorithms are still in their early stages of development, and improving their accuracy, speed, and ability to incorporate complex tissue modeling is required for the practical usage of this technology.

QPA methods consist of inverting both optical and acoustic propagation in photoacoustic imaging. The acoustic inversion can be implemented with several methods and requires accurate modeling of transducer response (such as directivity, bandwidth) and tissue realistic acoustic properties [10]. While acoustic inversion is crucial for QPA, the optical and acoustic processes can be decoupled and solved independently [2]. Accurate modeling of the light propagation is important in QPA and methods including simple models like Beer–Lambert law to Radiative Transfer Equation, Diffusion Approximation, and Monte Carlo were used [2], [9], [11]. After modeling light propagation, QPA methods use model fitting, iterative solutions, or minimization to retrieve optical properties [2], [9], [11]. The challenges in QPA imaging include the nontrivial nature of obtaining absolute chromophore concentrations from photoacoustic images obtained at multiple wavelengths, which is essential for accurate functional and molecular imaging [2]. The inverse problems involved in QPA imaging are nonlinear and ill-posed and often result in the non-uniqueness of solutions [9]. This work explores utilizing a known chromophore as a fluence marker to mitigate these issues.

We propose using known absorption coefficients of a reference chromophore as fluence markers to enhance QPA accuracy. The reference chromophore can be intrinsic absorbers like arterial oxygenated blood, exogenous contrast agents, catheters, or implants with known optical properties. Arterial blood is a natural choice, applicable in clinical scenarios where an artery is near the target tissue, such as imaging arterial wall atherosclerosis (using blood inside the lumen as a reference) or tumor imaging (using the feeding artery as a reference). We propose using a reference chromophore as prior information to improve QPA accuracy and convergence. Previous QPA fluence modeling has employed priors like average tissue scattering [9], ultrasound-based tissue surface data [3], [12], [13], implanted spectrally flat absorbers [14], catheters [15], and exogenous contrast agents [16]. These methods used the prior information for direct modeling of the fluence, which might pose challenges for deep tissue imaging as they lack feedback from the measured photoacoustic signal to refine fluence modeling. The concept of calibrating fluence using arterial blood was first introduced in a patent [17] and employed in oxygen saturation imaging [18]. This approach follows a two-step methodology: first, calibrating arterial fluence based on Beer–Lambert’s law, and then assuming a uniform fluence across nearby structures to estimate absorption. However, the assumption that fluence in a nearby vein (up to 10 mm) is identical to the calibrated arterial fluence simplifies the complex light interactions between the calibration marker and surrounding tissue, overlooking spatial variations. Key knowledge gaps in QPA using fluence markers include (I) accurately modeling fluence markers in optical inversions and (II) practically obtaining a fluence marker in complex tissues, highlighting the need for advanced and generic methods that consider the interaction between the unknown tissue and the fluence marker.

In this article, we introduce a novel approach to incorporate a known fluence marker into optical inversions for QPA, enhancing QPA accuracy and broadening its applicability across various optical inversions. We demonstrate spectral decoloring and magnitude recovery with the proposed method on experimental data. By embedding the fluence marker directly within optical modeling and parameter updates, we demonstrate its use in conventional QPA methods; simple iterative inversion, and a gradient-based QPA method. We systematically evaluate how the fluence marker improves QPA accuracy and spectral recovery and assess their performance in complex phantoms. Additionally, we highlight the practical relevance of arterial blood as a fluence marker in carotid plaque quantification.

## 2 Methods

In this section, we present the formulation of QPA and the optical inversion in Section 2.1, optical forward modeling, and QPA algorithms used for inversion in Section 2.4, respectively. Our approach to incorporating prior information from the reference chromophore is highlighted in the QPA algorithms. Section 2.6 describes the implementation and Section 2.7 includes the potential applications.

### 2.1 Quantitative photoacoustic imaging

In PA imaging, the tissue is illuminated with pulsed light. The light propagating through the tissue experiences attenuation throughout the medium due to absorption and scattering, resulting in a distribution, known as light fluence (ϕ). The energy absorbed by a chromophore at any position r is proportional to the fluence and the optical absorption of the chromophore, given by,

(1) $$H\left(r,\lambda \right)=\mu_{a}\left(r,\lambda \right)\phi \left(r,\lambda ,\mu_{a},\mu_{s}^{\prime }\right)$$

where H(r,λ) is the energy absorbed at a particular wavelength λ. The reduced scattering coefficient $\mu_{s}^{\prime }\left(r,\lambda \right)=\left(1-g\right)\mu_{s}\left(r,\lambda \right)$ and the absorption coefficient $\mu_{a}\left(r,\lambda \right)=C\left(r\right)\epsilon \left(r,\lambda \right)$ are again dependent on the wavelength λ and position r (omitted in Eq. (1) for convenience). Here, $\mu_{s}$ is the scattering coefficient, g is the anisotropy factor, C(r) is the concentration, and ϵ(r,λ) is the molar extinction coefficient. The energy absorbed by the chromophores results in a local rise in the temperature and pressure in the sample. The initial pressure rise is given by,

(2) $$p_{0}\left(r,\lambda \right)=\Gamma H\left(r,\lambda \right)$$

where Γ is the Grüneisen parameter, indicating PA efficiency. The instant pressure variation produces an acoustic wave that travels through the sample and can be measured at the boundary using an ultrasound transducer. The boundary signal $p_{t}\left(r_{s},\lambda \right)$ is used to reconstruct an approximation of the initial pressure distribution $\overset{ˆ}{p}\left(r,\lambda \right)$, called a PA image, where $r_{s}$ represents the transducer location. The photoacoustic image is proportional to the absorbed energy distribution H(r,λ). When Γ and the transducer characteristics are known or measured experimentally, H(r,λ) can be accurately obtained from $\overset{ˆ}{p}\left(r,\lambda \right)$. In this article, we assume the ideal acoustic detection and inversion, and a spatially invariant Γ, allowing accurate measurement of H(r,λ). Thus, we start the optical inversion from the measured absorbed energy. QPA imaging aims to retrieve the concentration of chromophores C(r) from H(r,λ). The absorbed energy spectrum at a point r is proportional to the absorption spectrum at that point and is reduced by the accumulated light absorption along the photon path. This modification of the original spectra by the spectrally varying absorption in the photon, known as spectral coloring, prevents the direct fitting of known spectra to absorbed energy spectra for estimating chromophore concentrations. An approach to retrieve the chromophore concentrations in QPA imaging is to use a light transport model and adjust parameters (absorption and scattering) in the corresponding forward problem until the simulated or modeled data matches the measured data.

### 2.2 Fluence marker assumption and its practical relevance

Although the fluence marker can be any known object, as we are interested in carotid plaque quantification we present the practical relevance of using arterial blood as a fluence marker. Blood’s optical properties are influenced by oxygen saturation (SpO_2_), hematocrit (Hct), and hemoglobin concentration, which can be accurately measured. Oxygen saturation is commonly assessed with pulse oximeters, while Hct is routinely measured in clinical settings in blood tests. Photoacoustic measurement at the isosbestic point of oxy and deoxy hemoglobin is independent of SpO_2_ levels. Using a pulse oximeter to measure the arterial oxygen saturation and a blood test before the imaging we can obtain information on these two variables. An FDA-approved pulse oximeter has an estimated maximum error of 4% and the error in Hct measurement has a maximum of 1% error. With SpO_2_ and Hct data, the absorption coefficient of blood in the carotid artery can be estimated reliably using models presented in [19]. The advantage of photoacoustic imaging is that we can obtain coregistered ultrasound images which helps in the spatial estimation of blood in the lumen of the carotid artery. This allows us to make a realistic estimation of the arterial blood spatial location and its optical properties, making blood a suitable fluence marker for this application.

### 2.3 Optical modeling

In this work, we utilize two light propagation models. First, we make a simplifying assumption by treating the unknown plaque as a homogeneous sample, allowing us to apply an exponentially decaying Beer–Lambert law for light propagation modeling.

(3) $$\phi \left(r\right)=\phi \left(0\right)e^{-\mu_{a}r}$$

Here, we model the fluence, ϕ(r), within the blood, specifically inside the lumen of the artery at a location r from the origin, chosen as the light entry point to the plaque. The average optical absorption coefficient, $\mu_{a}$, of the plaque tissue located above can be estimated as explained in Section 2.4. Here, $\phi_{0}$ denotes the fluence at the surface of the plaque.

For more accurate modeling with a heterogeneous plaque assumption we also make use of the diffusion approximation model. We use a 2D diffusion approximation for light propagation modeling to demonstrate the proposed method. Diffusion approximation can be written as,

(4) $$\mu_{a}\left(r,\lambda \right)\phi \left(r,\lambda ,\mu_{a},\mu_{s}^{\prime }\right)-\nabla \cdot \left(\kappa \left(r,\lambda \right)\nabla \right)\phi \left(r,\lambda ,\mu_{a},\mu_{s}^{\prime }\right)=q_{0}\left(r,\lambda \right)$$

where $\kappa =1/3\left(\mu_{a}+\mu_{s}^{\prime }\right)$ is the optical diffusion coefficient, and $q_{0}\left(r,\lambda \right)$ is the isotropic source term and $\mu_{s}^{\prime }\gg \mu_{a}$.

We employed the open-source NIRFAST software package for MATLAB (MathWorks, Inc., USA) to implement the diffusion approximation using FEM [20]. The area is discretized into finite elements, enabling the diffusion equation to be solved at discrete points with optical properties such as $\mu_{a}$, $\mu_{s}^{\prime }$ and the resultant fluence are represented as piecewise constant within each finite element. We used numerical phantoms with optical property maps assigned to image pixels. The FEM mesh is created from a Cartesian grid with diagonal edges forming triangular elements, directly assigning optical properties from phantom masks to mesh elements, avoiding extra interpolation. Light sources are isotropically distributed Gaussian sources placed one grid distance inside the boundary. The boundary condition is chosen such that the light can exit at the edge of the tissue, but cannot return [20], with refractive index n of the medium outside (set to 1 for air) and inside the boundary (set to 1.33 for tissue).

**Fig. 1:**
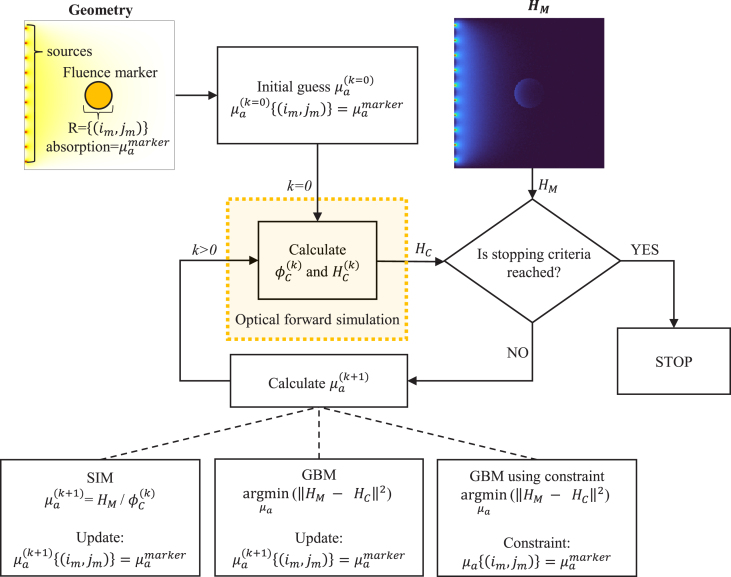
Fluence marker-based iterative optical inversion for quantitative photoacoustic imaging.

The forward operation uses GPU-based femdata_FD() from NIRFASTer [20]. The resultant fluence was obtained by combining fluence maps from each source using the superposition. The absorbed energy distribution was calculated as the product of fluence and the absorption coefficient. Finally, the measured absorbed energy distribution $H_{M}$, input to the optical inversion problem Fig. 1 , was modeled by adding Gaussian white noise at a specific SNR.

### 2.4 Optical inversion with fluence marker

The optical inversion scheme for recovering the absorption coefficient distribution employs a known reference absorber as a fluence marker, as illustrated in Fig. 1. The algorithm adjusts the absorption coefficient $\mu_{a}$ to minimize the difference between the measured absorbed energy distribution, $H_{M}$, and the model-calculated energy distribution, $H_{C}$. Known parameters include the source location, the fluence marker’s absorption coefficient, $\mu_{a}^{marker}$, and its location, $R=\left(i_{m},j_{m}\right)$, where $i_{m}$ and $j_{m}$ are row and column indices of the marker, respectively. The scattering distribution is assumed to be spatially invariant and known.

#### 2.4.1 Direct inversion of Beer–Lambert law with fluence marker

In the case of a homogeneous medium, one can invert the Beer–Lambert law. For example, if the fluence from the arterial blood is known, to estimate the fluence in the plaque, one can make a simplistic assumption of a homogeneous plaque and retrieve the fluence by simply inverting the Beer–Lambert law which matches the $H_{M}$ in the plaque region. When light passes through the fluence marker to reach the plaque we can obtain the unknown plaque absorption as,

(5) $$\mu_{a}^{unknown}e^{\mu_{a}^{unknown}}=\frac{H_{M}^{unknown}}{e^{r}H_{M}^{marker}}\mu_{a}^{marker}$$

Here $H_{M}^{marker}$ is the absorbed energy at the fluence marker (blood in the carotid artery) and $H_{M}^{unknown}$ is the absorbed energy at the unknown plaque sample, both these retrieved from the photoacoustic image to estimate the absorption coefficient ($\mu_{a}^{unknown}$) of the unknown plaque sample. The $\mu_{a}^{unknown}$ can be computed for pixels in the unknown chromophore by using the respective distances r, along the depth direction to the fluence marker. If we consider the fluence in the unknown chromophore to be the same as the marker, then Eq. (5) becomes,

(6) $$\mu_{a}^{unknown}=\frac{H_{M}^{unknown}}{H_{M}^{marker}}\mu_{a}^{marker}$$

We used these approaches to obtain the absorption coefficient of an unknown chromophore in spectrally varying phantoms in both simulation and experiment and used it to compare with the proposed fluence marker-based inversion method.

#### 2.4.2 Iterative inversion schemes with fluence marker

For a heterogeneous plaque assumption, we can have a generalized treatment using optical inversion schemes with the fluence marker assumption. We can use spatially resolved inversion schemes; (i) a simple iterative method (SIM) [21] and (ii) a gradient-based method (GBM) [22]. The algorithm begins by selecting an initial guess, $\mu_{a}^{\left(k=0\right)}$, with fluence marker elements set to the marker’s actual absorption coefficient. A forward optical simulation then estimates the fluence distribution, $\phi_{C}^{\left(k=0\right)}$, for this initial guess, and calculates the corresponding absorbed energy, $H_{C}^{\left(k=0\right)}$. A stopping criterion is checked, based on iteration count, a small error threshold for $\Delta H^{\left(k\right)}=H_{M}-H_{C}^{\left(k\right)}$, or a convergence point. If unmet, the absorption coefficient is further updated, repeating the process until convergence.

Both methods were adapted to incorporate the fluence marker for quantitative estimation. Each method minimizes the difference between $H_{M}$ and $H_{C}$. While the original GBM also estimated the scattering distribution [22], both methods here assume known scattering for consistency in comparison. The following sections detail our approach for recovering the absorption distribution using these fluence marker-enhanced techniques.

##### Recovery of absorption coefficient using SIM with fluence marker:.

The SIM iteratively updates the absorption coefficient $\mu_{a}$ to minimize $\Delta H^{\left(k\right)}$. Each update relies solely on the element-wise ratio of $H_{M}$ to the fluence derived from the current $\mu_{a}$ distribution, making this approach computationally efficient. Algorithm 1 provides pseudocode for estimating $\mu_{a}$ using the fluence marker.

**Figure:**
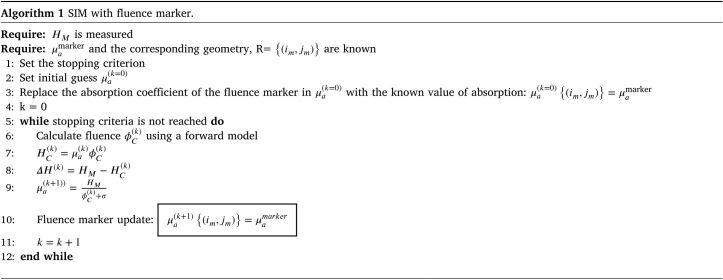

A regularization parameter σ is introduced to stabilize regions with low fluence [21].

##### Recovery of absorption coefficient using GBM with fluence marker:.

The core of the gradient-based technique is to provide a functional gradient within an optimization algorithm for iterative minimization. The algorithm estimates an optimal value for $\mu_{a}$ to minimize the error. The functional gradient of the error, $\epsilon =\frac{1}{2}\int {\left(H_{M}-H_{C}\right)}^{2}dV$, is obtained by differentiating with respect to $\mu_{a}$ and is given by

(7) $$\frac{\partial \epsilon }{\partial \mu_{a}}=-\phi \left(H_{M}-H_{C}\right)+\phi \phi^{*}$$

where $\phi^{*}$ is the solution of the adjoint equation,

(8) $$\mu_{a}\phi^{*}+\nabla \cdot \left(\kappa \nabla \right)\phi^{*}=\mu_{a}\left(H_{M}-H_{C}\right)$$

For simplicity, the dependence of variables on position and wavelength is omitted. The term $\phi^{*}$ in Eq. (7) can be calculated using the same forward model but with the source term $\mu_{a}\left(H_{M}-H_{C}\right)$.

In our case, the value of $\mu_{a}$ is known at the location of the fluence marker, while $\mu_{a}$ at other points must be optimized, keeping the known values fixed. This can be approached in two ways: the first method includes an extra step to update $\mu_{a}$ at $\left\{\left(i_{m},j_{m}\right)\right\}$ after each iteration. The pseudocode for recovering the optical absorption distribution using GBM is presented in Algorithm 2.

**Figure:**
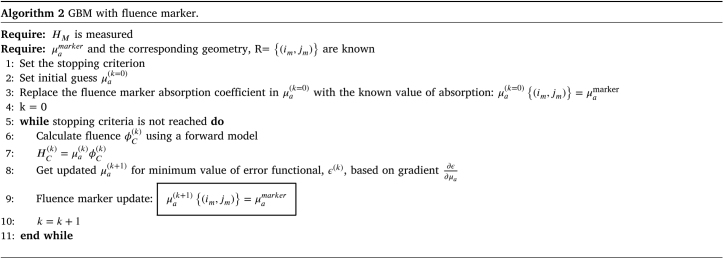

The second approach to incorporating the fluence marker in the GBM algorithm is to constrain the $\mu_{a}$ of the reference chromophore to remain unchanged during optimization. A spatial equality constraint enforces this, as detailed in the pseudocode in Algorithm 3.

**Figure:**
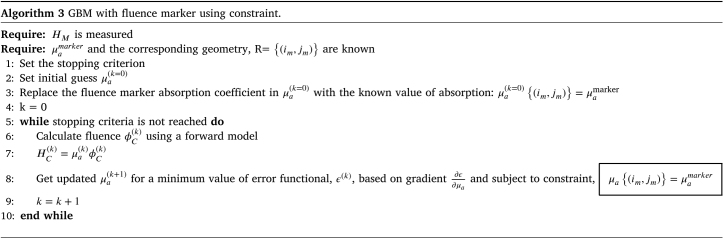

We used an equality constraint of the form $A_{eq}X=B_{eq}$, where X is the unknown $\mu_{a}$. In iterative solving, the unknown optical absorption map is constrained by the prior information of the fluence marker. To do so, the spatial equality constraint $A_{eq}X=B_{eq}$ can be imposed on the optical absorption by specifying all $a_{eq\left(i_{m},j_{m}\right)}=1$ and $b_{eq\left(i_{m},j_{m}\right)}=\mu_{a}^{marker}$, where $a_{eq}$ and $b_{eq}$ are the elements of $A_{eq}$ and $B_{eq}$ and $i_{m}$ and $j_{m}$ are the indices corresponding to the fluence marker location.

### 2.5 Experimental demonstration

**Fig. 2:**
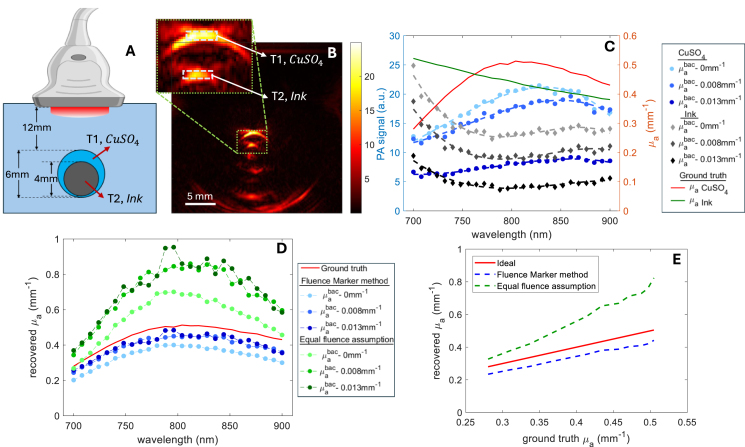
(A) Experimental setup and (B) reconstructed PA image for λ=800nm. (C) The average photoacoustic signal obtained for India Ink and CuSO${}_{4}$ at different wavelengths compared to the ground truth absorption spectra. Recovered absorption spectra using (D) proposed fluence marker-based optical inversion method compared to equal fluence assumption and the ground truth absorption spectra. (E) Predicted absorption coefficient plotted against ground truth.

To simulate the geometry of a carotid artery with plaque, we used two concentric polyurethane tubes (Raumedic AG, Germany) with inner diameters of 6 mm and 4 mm with 0.1 and 0.15 mm thickness respectively, as shown in Fig. 2. The inner tube (T2), acting as a fluence marker, was filled with India Ink (Talens, The Netherlands), which has an absorption coefficient of $0.44\;{\text{mm}}^{-1}$ at 800 nm. The outer tube (T1), representing an unknown chromophore, was filled with ${\text{CuSO}}_{4}\cdot 5{\text{H}}_{2}\text{O}$ (Sigma-Aldrich, USA) with an absorption coefficient of $0.51\;{\text{mm}}^{-1}$ at 800nm. The tubes were placed in a tank with an absorbing and scattering medium. The surrounding medium in the tank was initially prepared with 3% intralipid in water, followed by two different concentrations of ${\text{CuSO}}_{4}$ 6.8 mM and 11.61 mM, which corresponds to the absorption coefficient values of 0.0076 ${\mathrm{mm}}^{-1}$ and 0.0131 ${\mathrm{mm}}^{-1}$ respectively at 800nm were added to incrementally adjust the absorption in the medium.

A tunable diode-pumped OPO laser (Spitlight EVO-OPO, 100 Hz PRF, 5 ns pulse width, Innolas GmbH, Germany) was used for sample illumination, with the beam delivered through an 8 mm diameter optical fiber bundle. Photoacoustic (PA) data were recorded using a Verasonics ultrasound system (Vantage 256, Kirkland, WA, United States) equipped with a linear transducer (L12-3v) with a center frequency of 7 MHz. PA images were captured under varying ${\text{CuSO}}_{4}$ concentrations in the background medium.

For the optical inversion of PA image, the values $H_{M}^{unknown}$ and $H_{M}^{marker}$ were determined from the PA signal from T1 and T2 (as shown in Fig. 2) by assuming Γ=1. Then the absorption coefficient of unknown chromophore (${\text{CuSO}}_{4}$) was obtained by optimizing Eq. (5) using MATLAB’s fsolve function with initial guess $1\times 10^{-5}\;\mathrm{mm}$^−1^) with the calculated distance to the lumen of the inner tube as r and the known values $\mu_{a}^{marker}$ of the fluence marker.

The images were acquired across wavelengths from 700 to 900 nm, normalized to pulse energy, and analyzed for the spectral characteristics of both tubes. The ground truth absorption coefficient spectra were obtained using a spectrophotometer (Agilent Cary 3500, California, United States) through absorbance measurements, under the assumption that both Indian Ink and ${\text{CuSO}}_{4}$ solutions are non-scattering. We analyzed the spectral coloring effect introduced by the medium and compared it with the proposed fluence marker-based optical inversion presented in Section 2.4.1. Additionally, we compared our results with an estimation based on the equal fluence assumption, where the fluence in ${\text{CuSO}}_{4}$ is assumed to be equivalent to that of the fluence marker (Indian Ink).

### 2.6 Implementation of iterative inversion

**Fig. 3:**
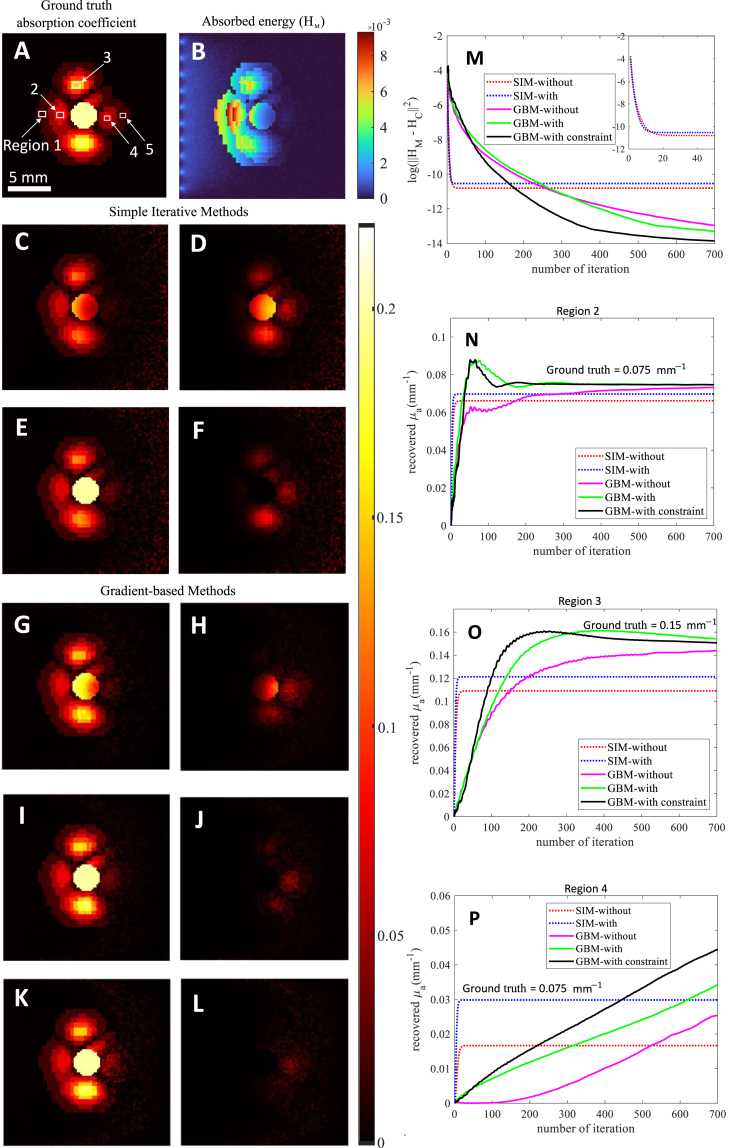
Comparison of Algorithms: (A) ground truth absorption coefficient (mm^−1^) distribution and (B) absorbed energy (mm^−2^s^−1^) obtained using forward optical simulation. (C)-(F) Comparison of reconstructed images using SIM: (C) recovered $\mu_{a}$ (mm^−1^), and (D) absolute error (difference of ground truth and recovered $\mu_{a}$) without fluence marker and (E) recovered $\mu_{a}$ and (F) error with fluence marker. (G)-(L) Comparison of reconstructed images using GBM: (G) recovered $\mu_{a}$ and (H) error without fluence marker, (I) recovered $\mu_{a}$ and (J) error with fluence marker, and (K) recovered $\mu_{a}$, and (L) error with fluence marker using constraint. (M) Log of the sum of the squared error against iteration number and the nature of convergence for SIM methods for the first 50 iterations (inset). The average value of the absorption coefficient updates against iterations in different regions of the reconstructed image: (N) Region 2, (O) Region 3, (P) Region 4.

The fluence marker concept integrates a known chromophore in the QPA algorithm. Here, phantoms with a central circular disc represent an artery cross-section as a fluence marker, with chromophore absorption coefficients modeling tissue properties in the NIR region [23] and a constant scattering coefficient of 2 mm^−1^. Optical properties are mapped to the mesh by placing FEM nodes at pixel centers. Simulations use ten point sources placed just inside the boundary. The absorbed energy density for each $\mu_{a}$ distribution was computed with NIRFAST (Section 2.3), adding white Gaussian noise (SNR = 30 dB) by measuring the signal power using MATLAB’s awgn function. This energy distribution served as the input $H_{M}$ for inversion. All programs used the same initial guess for $\mu_{a}$ ($1\times 10^{-10}\;\mathrm{mm}$^−1^) and a known scattering coefficient. The choice of initial guess has no significant impact on the results provided it is sufficiently small (on the order of $\leq 10^{-5}$). For SIM, the regularization parameter σ was set to 0.002. Fluence marker pixels are set to the marker’s actual absorption coefficient. The forward simulation then calculates $\phi_{C}^{\left(k\right)}$ and $H_{C}^{\left(k\right)}$. The $\mu_{a}$ update is derived from the ratio of $H_{M}$ to $\phi_{C}^{\left(k\right)}$ from the previous iteration. Before each new iteration, marker elements in $\mu_{a}$ are reset to the original value. Iterations continue until stopping criteria are met. The GBM algorithm, using MATLAB’s fminunc, minimizes the error function with the supplied gradient. After calculating $\phi_{C}^{\left(k\right)}$ and $H_{C}^{\left(k\right)}$, $\mu_{a}$ is updated to minimize error, with marker elements reset before the next iteration. This process repeats until convergence. In constrained GBM, MATLAB’s fmincon with equality constraint $A_{eq}X=B_{eq}$ is used. Here, $A_{eq}$ and $B_{eq}$ are specified, and no post-optimization update is required, as the constraint maintains the fluence marker. Using a complex spatially varying phantom (Fig. 3A) inspired by Ref. [22], we compared methods with and without the marker. This 20 mm × 20 mm phantom (pixel size 0.2 mm) has a central fluence marker (absorption 0.2 mm^−1^) with nine absorption levels ranging from 0.001 mm^−1^ to 0.2 mm^−1^. We applied five reconstruction methods: (i) SIM (ii) SIM with fluence marker 1 (iii) GBM (iv) GBM with fluence marker 2, and (v) GBM with fluence marker using constraints 3, each iterated 700 times. At each iteration, any negative values in the updated $\mu_{a}$ resulting from noise were set to zero. To assess image quality, we used PSNR, excluding the fluence marker region from $\mu_{a}$ calculations.

For further analysis, we compared convergence and accuracy for five regions in the phantom, plotting the average recovered $\mu_{a}$ across iterations to track recovery progress for each method (Fig. 3).

### 2.7 Application

We evaluated the proposed method’s ability to mitigate spectral coloring. A simple digital phantom with two concentric discs, representing a cross-section of the carotid artery surrounded by plaque, was used. The phantom had dimensions of 20 mm × 20 mm and a resolution of 0.2 mm. Blood served as the fluence marker, while the surrounding chromophore represented plaque with unknown optical properties. The optical absorption of the unknown chromophore and background varied with wavelength, whereas the fluence marker’s absorption (0.2 mm^−1^) was considered invariant. The absorption coefficient of the unknown chromophore was recovered using a GBM with the fluence marker method across multiple wavelengths. The absorption spectrum was also retrieved by assuming equal fluence within the unknown region and the fluence marker (Eq. (6)). We then compared the accuracy of the proposed method and the equal fluence assumption in addressing spectral coloring.

We applied the method to a carotid artery cross-section with realistic tissue properties, inspired by [24]. The phantom, representing artery and surrounding tissue (24 mm × 24 mm, 0.15 mm resolution), assigned optical properties based on literature values [19], [23], [25], [26] at λ=930nm, where lipid absorption peaks (Table 3). The forward simulation provided the absorbed energy density, with Gaussian noise (SNR = 40 dB) added. We have compared GBM with and without the fluence marker for a fixed number of iterations (1000) and compared it against the ground truth. To assess practical applicability, we examined fluence marker uncertainties. Hct measurement variability is under 1% in clinical practice [27], and FDA-approved pulse oximeters show a maximum 4% error. For robustness testing, a 10% error margin was simulated, introducing a 10% increase and decrease in the fluence marker’s absorption to evaluate the effects on chromophore recovery.

## 3 Results

In this section, we first present the advantage of using a fluence marker experimentally in optical inversion. Then we compare different optical inversion methods and present application of fluence marker in carotid plaque imaging.

Fig. 2B displays the photoacoustic image of two tubes acquired at 800 nm. The photoacoustic spectra in Fig. 2C reveal the spectral variations in both the CuSO${}_{4}$ and India Ink spectra, comparing them to the ground truth absorption spectra. In the CuSO${}_{4}$ spectrum, a peak shift is observed from its original position at 810 nm. As absorption in the surrounding medium increases, this red shift in the CuSO${}_{4}$ spectrum becomes more pronounced, accompanied by a reduction in spectral magnitude. Notably, the CuSO${}_{4}$ spectrum affects the India Ink spectrum, resulting in elevated PA signals between 700–750 nm and 850–900 nm, which distorts the spectral profile of India Ink from the ground truth spectrum.

Utilizing the proposed fluence marker-based optical inversion (Fig. 2D), both spectral shape and magnitude were retrieved. If we consider an equal fluence assumption in the surrounding medium (Fig. 2D), the spectral shape is recovered to a reasonable level, but shows error in the magnitude estimation. The optical absorption of the surrounding medium in the tank was modified in the experiment; hence, the recovered spectra for all three cases after the optical inversion are expected to align with the original spectra. The recovered spectrum using the fluence marker method in Fig. 2D closely matches the ground truth spectrum, with minor discrepancies attributed to unaccounted optical scattering effects of the chromophore. With the proposed fluence marker-based optical inversion a mean error in the optical absorption coefficient estimate of $0.07\;{\mathrm{mm}}^{-1}$ standard deviation of $0.013\;{\mathrm{mm}}^{-1}$ was observed. The estimation using an equal fluence assumption resulted in a mean absorption coefficient error of $0.21\;{\mathrm{mm}}^{-1}$ with a standard deviation of $0.079\;{\mathrm{mm}}^{-1}$. These experimental results demonstrate the capability of the proposed approach in accurately quantifying chromophores. The spectral recovery also confirms that an equal fluence assumption around the fluence marker can result in quantification error as shown in Fig. 2E. In this analysis, we assumed a homogeneous unknown tissue for the inversion. In the following results, we will demonstrate and compare optical inversion schemes for heterogeneous media using the fluence marker.

Fig. 3 compares the recovered absorption coefficient ($\mu_{a}$) using SIM and GBM algorithms, with and without the fluence marker. Fig. 3A shows the digital phantom, and Fig. 3B shows the absorbed energy distribution ($H_{M}$). Recovered $\mu_{a}$ and absolute error for SIM without and with the fluence marker are presented in Figs. 3C-F. For GBM, recovered $\mu_{a}$ and error appear in Figs. 3G-J, with and without the fluence marker, and Figs. 3K-L display results for GBM with the fluence marker using constraint optimization. Fig. 3M shows error ($\log\left({\left\|H_{M}-H_{C}\right\|}^{2}\right)$) convergence over iterations and Fig. 3N-P shows the average value of the absorption coefficient updates against iterations in different regions of the reconstructed image.

The recovered $\mu_{a}$ distribution using SIM shows accurate reconstruction at shallow depths (< 10 mm), with reduced accuracy at greater depths. Comparing Figs. 3D and 3F and PSNR values in Table 1, SIM with the fluence marker improves reconstruction. GBM offers better noise reduction and accuracy, with further improvement when using the fluence marker. The GBM with fluence marker constraint yields the highest accuracy, as shown in Fig. 3L. SIM converges within 25 iterations but reaches a higher final error of −10.54 mm^−2^s^−1^ with the fluence marker due to background noise, compared to −10.80 without. GBM with the fluence marker shows oscillations and requires over 50 iterations to stabilize. GBM without the marker, converges to an error of −12.97 after 700 iterations. Using the fluence marker constraint, GBM achieves the same error level in only 348 iterations.

**Table 1:** Comparison of PSNR values of recovered images using different methods.

Method	PSNR
	(dB)
SIM	37.53
SIM with fluence marker	39.27
GBM	43.24
GBM with fluence marker	45.47
GBM with fluence marker using constraint	46.37

Table 2 summarizes the recovered $\mu_{a}$ across regions. SIM with the fluence marker improves $\mu_{a}$ accuracy across all regions despite higher background error, while GBM with the fluence marker and constraint yields the most accurate recovery, especially at depths over 10 mm (Regions 4 and 5). Region 4 shows a trend toward true $\mu_{a}$ with the fluence marker in GBM. Extended iterations in Region 4 allowed only GBM with constraints to converge close to the true $\mu_{a}$ value ($0.08\;{\mathrm{mm}}^{-1}$, after 1366 iterations) before halting when the function no longer decreased within the optimality tolerance. At greater depths (Region 5), noise impacts $\mu_{a}$ recovery, and none of the methods recovered absorption values accurately, primarily due to the shadowing of this region with the high absorption of the fluence marker.

**Table 2:** Comparison of recovered $\mu_{a}$ (in mm^-1^) in Fig. 3 after 700 iterations using different reconstruction methods. The corresponding error in percentage (%) is given in brackets.

Region	Ground truth $\mu_{a}$	Recovered $\mu_{a}$ using SIM		Recovered $\mu_{a}$ using GBM		
		Without marker	With marker	Without marker	With marker	With marker-constraint
1	0.020	0.0195 (2.5)	0.0196 (2)	0.0199 (0.5)	0.020 (0)	0.020 (0)
2	0.075	0.0661 (11.8)	0.0697 (7)	0.0732 (2.5)	0.0746 (0.5)	0.0747 (0.4)
3	0.150	0.1090 (27.3)	0.1212 (19.2)	0.1437 (4.2)	0.1541 (2.7)	0.1506 (0.4)
4	0.075	0.0167 (77.7)	0.0298 (60.3)	0.0253 (66.3)	0.0342 (54.4)	0.0445 (40.6)
5	0.001	0.0037 (270)	0.0053 (430)	0.0001 (90)	0.0037 (270)	0.0035 (250)

Key observations from Table 2 and Fig. 3C-P include: (i) GBM offers better noise reduction and accuracy than SIM but requires more iterations, (ii) the fluence marker improves both algorithms and (iii) accuracy decreases with depth. Algorithm ranking by accuracy and iterations is as follows: GBM with fluence marker constraint, GBM with fluence marker, GBM without fluence marker, SIM with fluence marker, and SIM without fluence marker.

**Fig. 4:**
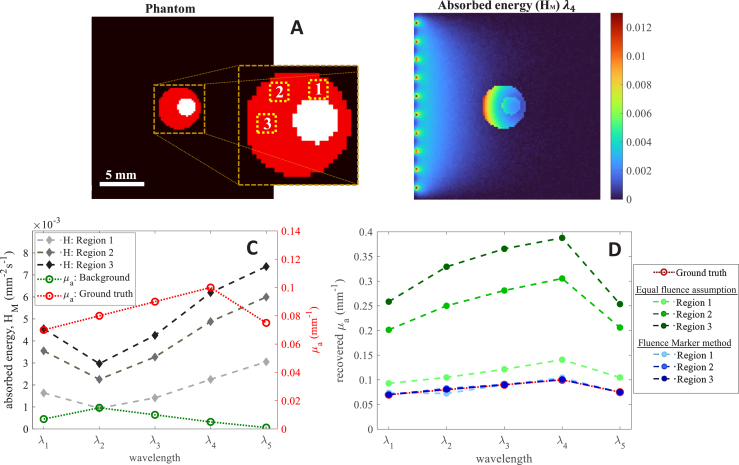
Spectral imaging : (A) Digital phantom used for simulations and the inset is a magnified image of the sample where the area of averaging is marked. (B) Absorbed energy distribution for wavelength $\lambda_{4}$. (C) Average absorbed energy for different regions in the unknown chromophore and ground truth $\mu_{a}$ of unknown chromophore and background of sample for five different wavelengths. (D) Comparison of the absorption spectrum of the unknown chromophore at different regions recovered using fluence marker method and equal fluence assumption.

The proposed method was tested for quantitative recovery of $\mu_{a}$ in multi-wavelength imaging. Fig. 4A shows the carotid artery phantom, where the central circular disc mimics the arterial lumen as the fluence marker, surrounded by plaque (unknown chromophore) within a uniform background. Fig. 4B illustrates the absorbed energy distribution at $\lambda_{4}$. Fig. 4C displays both the ground truth $\mu_{a}$ values for the background and the unknown chromophore, along with the absorbed energy in the three regions marked in Fig. 4A across five wavelengths.

We employed GBM with a fluence marker for the optical inversion and compared this approach to the equal fluence assumption, as shown in Fig. 4D. For the GBM method, a maximum of 450 iterations was set for reconstruction. The average $\mu_{a}$ at each region using both the GBM with fluence marker and the equal fluence assumption is presented in Fig. 4D. For Region 1, the equal fluence assumption yielded an estimate close to the actual value, while for Regions 2 and 3, an overestimation of $\mu_{a}$ occurred. This overestimation in absorption is attributed to the fluence variation with distance from the reference chromophore. In contrast, the fluence marker-based inversion effectively recovered the spectral shape and magnitude of $\mu_{a}$ across regions. These findings align with experimental data (Fig. 2D), where overestimation is similarly observed under the equal fluence assumption. This emphasizes the importance of fluence marker-based inversion for accurate recovery of both spectral shape and magnitude, particularly in heterogeneous environments.

**Fig. 5:**
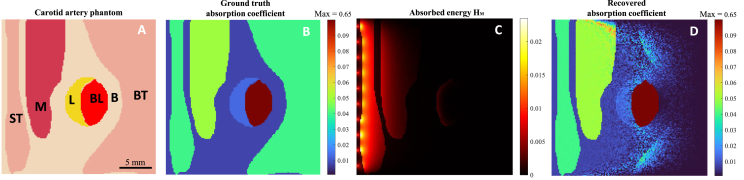
(A) Digital phantom used for the simulation (ST: superficial tissue, M: muscle, L: lipid, BL: blood, B: background, BT: background tissue). (B) Ground truth absorption coefficient (mm^−1^) distribution (see Table 3) and (C) absorbed energy (mm^−2^s^−1^) obtained using forward optical simulation. (D) Recovered $\mu_{a}$ distribution. Note that the range of the absorption coefficient map in (B) and (D) are from 0 to 0.2 mm^−1^, while the maximum value of the blood (fluence marker) is 0.65 mm^−1^.

A potential application of the fluence marker technique for carotid artery imaging is shown in Fig. 5. Fig. 5A presents the digital phantom, while Fig. 5B shows the ground truth $\mu_{a}$ distribution, and Fig. 5C displays the absorbed energy density. The recovered $\mu_{a}$ using GBM with fluence marker update is shown in Fig. 5D. Table 3 lists ground truth $\mu_{a}$, corresponding pixel values, and average recovered $\mu_{a}$ for each phantom component. The average $\mu_{a}$ was calculated by averaging pixels for each component. As seen in Fig. 5D and Table 3, the proposed method accurately recovered lipid $\mu_{a}$ using blood as a fluence marker, with less than 1% error, and reconstructed superficial tissue and muscle effectively. Due to the absorption by muscle and tissue, light intensity is minimal at deeper regions, resulting in noise in the recovered $\mu_{a}$ at depths > 12 mm, especially in the background tissue at 15 mm, where the error is highest.

Assuming a ±10% error in the fluence marker’s absorption estimation in a practical setting is presented in Table 3. Table shows that all components, except lipid were recovered accurately. Lipid $\mu_{a}$ varied by approximately 15%, indicating how quantification of chromophores near the fluence marker can affect the estimation. The $\mu_{a}$ recovery without the marker yielded lower accuracy across all components. It should be noted that for lipid, the resulting error is 36% without the fluence marker, compared to 15% with the fluence marker. The GBM algorithm without a marker required 4,223 iterations to converge, accurately recovering the muscle, superficial tissue, and background but yielding a 12% error in lipid absorption. In contrast, the GBM with a marker converged in only 1,663 iterations, accurately recovering all components except for the background tissue. This comparison shows that even an approximation estimate of the fluence marker can improve the optical inversion.

**Table 3:** Comparison of the recovered values of the absorption coefficients, $\mu_{a}$ (in mm^-1^) for different components of the phantom in Fig. 5: recovered with fluence marker, recovered when a ±10% error is included in the absorption of the marker and recovered without the fluence marker. $\mu_{a}^{M}$ is the absorption coefficient of the marker, $\mu_{a}^{GT}$ is the ground truth absorption coefficient. The corresponding error in percentage (%) is given in brackets.

Component	$\mu_{aGT}$	Recovered $\mu_{a}$			
		$\mu_{a}^{M}=\mu_{a}^{GT}$	$\mu_{a}^{M}=\mu_{a}^{GT}$+10%	$\mu_{a}^{M}=\mu_{a}^{GT}$-10%	Without Marker
Blood (BL)	0.650	NA	NA	NA	0.104 (84)
Lipid (L)	0.013	0.013 (0.77)	0.015 (14.5)	0.012 (10.8)	0.008 (36.2)
Muscle (M)	0.050	0.051 (2)	0.051 (2)	0.049 (2)	0.049 (2.4)
Superficial tissue (ST)	0.040	0.040 (0)	0.040 (0)	0.040 (0)	0.039 (0.25)
Background tissue (BT)	0.040	0.006 (85)	0.004 (90.5)	0.004 (91.3)	0.002 (94.2)
Background (B)	0.008	0.008 (2.5)	0.008 (1.3)	0.008 (3.8)	0.005 (32.5)

## 4 Discussion

We introduced the use of a known chromophore’s absorption coefficient as a fluence marker in optical inversion for the quantitative recovery of chromophores from photoacoustic (PA) images. Our results demonstrate that this approach enhances the accuracy of optical property quantification by anchoring the fluence at the fluence marker location.

Spectral coloring poses a challenge in quantitative photoacoustic (QPA) imaging, especially in cases with wavelength-dependent background absorption. The results in Fig. 4, Fig. 2 illustrate the impact of spectral coloring in environments with wavelength-dependent background absorption. The recovered spectrum under the equal fluence assumption suggests that this assumption holds true in regions at the same depth and in close proximity to the fluence marker. However, in regions at varying depths, even closer to the reference, the equal fluence assumption leads to inaccuracies. Specifically, both Fig. 4, Fig. 2 reveal an overestimation of the absorption coefficient in regions located above the fluence marker. The proposed fluence marker technique successfully recovered the absorption spectrum in terms of both spectral shape and absolute magnitude, independent of spatial position (see Fig. 4D). Notably, a consistent trend in spectral coloring and decoloring was observed with the proposed method in both simulation and experimental settings, validating the effectiveness and practical applicability of our method. These results underscore the potential of the fluence marker-based inversion technique for accurate chromophore quantification in complex, heterogeneous media.

The comparison of QPA algorithms in Fig. 3 showed that both SIM and GBM improved accuracy with fluence marker. The absolute error in recovered $\mu_{a}$ was reduced by up to 24%. The gradient-based method (GBM) with the fluence marker constraint resulted in the lowest absolute error (Fig. 3), while the simple iterative method (SIM) converged faster but had larger errors. Though GBMs required more iterations, they produced lower errors than SIM. The fluence marker reduced iterations needed in both methods, though constraint optimization remains computationally intensive. Region-wise analysis (Fig. 3) reveals accuracy is depth-dependent: at depths < 10 mm, the fluence marker offers minimal improvement (0.5%), while at depths of 10–20 mm, it enhances SIM by 17.5%, GBM by 9.4%, and GBM with constraint by 24.4%. Thus, the fluence marker improves QPA accuracy at greater depths, with GBM offering the best balance of accuracy and computational demand.

The carotid artery imaging application in Fig. 5 demonstrates the effectiveness of the fluence marker technique for recovering a lipid plaque target, with blood in the lumen serving as the fluence marker. This method recovers plaques with lower optical absorption, even when obscured by highly absorbing muscle and superficial tissue. Our analysis of fluence marker estimation errors suggests that even approximate, practically obtainable information can significantly improve chromophore absorption recovery.

The current implementation presents some limitations that should be acknowledged. In the experimental results shown in Fig. 2, a homogeneous tissue assumption was adopted. Conversely, for the heterogeneous case, we relied on simulated phantom data. These simulations assume ideal acoustic reconstruction, excluding practical artifacts like those seen in band-limited transducers, as observed in Fig. 2B. To enhance practical applicability, incorporating realistic transducer properties into the inversion process or deconvolving the transducer’s response is required. The estimation of the reduced scattering coefficient using a GBM is feasible, it was not included in this study. Furthermore, the simulations were conducted in 2D phantoms to reduce computational complexity to demonstrate the concept. However, extending the model to 3D would more accurately represent realistic scenarios and is a logical progression for future work.

The proposed fluence marker-based quantitative PA method has other potential applications in clinical settings with identifiable chromophores, such as tissue oxygenation using an artery as a reference, tumor imaging with feeding vessels, or targeting tissue near catheters or implants. It may also benefit optical wavefront shaping, diffuse optical imaging, and spectroscopy.

## 5 Conclusion

This study demonstrates, using both experimental and simulated phantoms, that integrating the optical properties of a known chromophore as a fluence marker in optical inversion can substantially enhance the accuracy of tissue quantification in photoacoustic imaging. The results show accurate recovery of spectral shape and magnitude in experimental settings. Comparisons across inversion methods indicate improvements of up to 17.5% in the simple iterative approach and 9.4% in the gradient-based approach when incorporating the fluence marker. Additionally, constrained optimization further boosted gradient-based accuracy by 24.4%. The fluence marker technique is effective in media with biologically relevant absorption and scattering properties, demonstrating potential in applications such as carotid plaque quantification. With further inclusion of acoustic transducer characteristics, this method could expand its utility for tissue quantification in various clinical contexts.

## CRediT authorship contribution statement

**Anjali Thomas:** Writing – review & editing, Writing – original draft, Visualization, Validation, Software, Methodology, Investigation. **Max Rietberg:** Writing – review & editing, Writing – original draft, Validation, Software, Methodology. **Mervenur Akkus:** Methodology. **Gijs van Soest:** Writing – review & editing, Supervision. **Kalloor Joseph Francis:** Writing – review & editing, Supervision, Project administration, Methodology, Investigation, Funding acquisition, Conceptualization.

## Funding

This work has received financial support from the Dutch Research Council (NWO), The Netherlands , for projects NWO-VENI (19165) and NWO-XS (OCENW.XS22.2.160).

## Declaration of competing interest

The authors declare the following financial interests/personal relationships which may be considered as potential competing interests: Gijs van Soest reports a relationship with Kaminari Medical BV that includes: consulting or advisory and equity or stocks. If there are other authors, they declare that they have no known competing financial interests or personal relationships that could have appeared to influence the work reported in this paper.

## Data Availability

Data will be made available on request.
